# ﻿A DNA barcode library for katydids, cave crickets, and leaf-rolling crickets (Tettigoniidae, Rhaphidophoridae and Gryllacrididae) from Zhejiang Province, China

**DOI:** 10.3897/zookeys.1123.86704

**Published:** 2022-10-05

**Authors:** Yizheng Zhao, Hui Wang, Huimin Huang, Zhijun Zhou

**Affiliations:** 1 Key Laboratory of Zoological Systematics and Application of Hebei Province, College of Life Sciences, Hebei University, Baoding, Hebei 071002, China Hebei University Baoding China; 2 Institute of Life Science and Green Development, Hebei University, Baoding, Hebei 071002, China Hebei University Baoding China

**Keywords:** Barcode Index Number, cryptic species, Ensifera, Orthoptera, species delimitation

## Abstract

Barcode libraries are generally assembled with two main objectives in mind: specimen identification and species discovery/delimitation. In this study, the standard COI barcode region was sequenced from 681 specimens belonging to katydids (Tettigoniidae), cave crickets (Rhaphidophoridae), and leaf-rolling crickets (Gryllacrididae) from Zhejiang Province, China. Of these, four COI-5P sequences were excluded from subsequent analyses because they were likely NUMTs (nuclear mitochondrial pseudogenes). The final dataset consisted of 677 barcode sequences representing 90 putative species-level taxa. Automated cluster delineation using the Barcode of Life Data System (BOLD) revealed 118 BINs (Barcodes Index Numbers). Among these 90 species-level taxa, 68 corresponded with morphospecies, while the remaining 22 were identified based on reverse taxonomy using BIN assignment. Thirteen of these morphospecies were represented by a single barcode (so-called singletons), and each of 19 morphospecies were split into more than one BIN. The consensus delimitation scheme yielded 55 Molecular Operational Taxonomic Units (MOTUs). Only four morphospecies (*I*_max_ > DNN) failed to be recovered as monophyletic clades (i.e., *Elimaeaterminalis*, *Phyllomimusklapperichi*, *Sinochloraszechwanensis* and *Xizicushowardi*), so it is speculated that these may be species complexes. Therefore, the diversity of katydids, cave crickets, and leaf-rolling crickets in Zhejiang Province is probably slightly higher than what current taxonomy would suggest.

## ﻿Introduction

Accurate specimen identification and species discovery are fundamental to taxonomic research and essential prerequisite for many fields of research such as ecology, biogeography, and conservation biology (Agapow et al. 2004; Collins and Cruickshank 2013). DNA barcoding using a standardized gene region (5´ region of the mitochondrial gene Cytochrome *c* oxidase subunit I, COI-5P) provide a powerful tool for specimen delimitation (Hebert et al. 2003). It can quickly distinguish species even with high morphological similarity, and it identifies cryptic genetic lineages within species, but it can fail if lineage sorting is incomplete (Yassin et al. 2009; Asis et al. 2016; Anderson et al. 2020). Specimen identification based on DNA barcodes does not rely on taxonomic expertise and can exclude the influence of human subjectivity in traditional morphological taxonomy. In recent years, increasing taxonomic practices have involved both morphological traits and DNA barcodes (DeSalle et al. 2005; Collado et al. 2021; Sabatelli et al. 2021). DNA barcodes have gained wide adoption for animal cryptic species recognition, species discovery, taxonomic revisions, and faunal assessments (Hebert et al. 2004, Tembe et al. 2014, Lone et al. 2020).

Cryptic species generally refer to highly genetically differentiated, but morphologically indistinguishable species (Van Campenhout et al. 2015). The discovery of cryptic species was critical for assessing biodiversity (Kundu et al. 2019). In the last 20 years, numerous studies using DNA barcoding have revealed cryptic species in several insect groups, such as Lepidoptera (Schonrogge et al. 2002; Burns et al. 2008), Thysanoptera (Tyagi et al. 2017), Diptera (Gajapathy et al. 2016; Chan-Chable et al. 2019). In morphological stasis, cryptic species within a complex or sister group remain highly morphologically similar for long periods of time, even tens of millions of years (Struck et al. 2017). Cryptic species may represent morphological stasis among related species experiencing similar environment conditions, but it may also reflect frequent, recent and/ or rapid speciation (Cerca et al. 2020).

Effective identification of a query specimen through DNA barcode sequence requires reliable reference libraries of known taxa. The process of assembling comprehensive and high-quality reference libraries of DNA barcodes allows the identification of newly collected specimens and accelerates taxonomic progress. The use of DNA barcoding for specimen identification and species discovery is greatly facilitated by the Barcode of Life Data System (BOLD, http://www.boldsystems.org).

Members of the suborder Ensifera diverged into grylloid (crickets) and non-grylloid (katydids) clades at the Triassic/Jurassic boundary (Zhou et al. 2017). Katydids (Tettigoniidae), cave crickets (Rhaphidophoridae), and leaf-rolling crickets (Gryllacrididae) of non-grylloid (katydids) clades constitute a nearly cosmopolitan group with up to 10,000 valid species (Cigliano et al. 2021). DNA barcoding studies on katydid and related ensiferan groups have increased recently (Guo et al. 2016; Hawlitschek et al. 2017; Zhou et al. 2019; Kim et al. 2020), which has led to about 15% (1449 species) having been barcoded (www.boldsystems.org), including 7841 public records belonging to 1058 Barcode Index Numbers (BINs) or 871 species from Tettigoniidae, 145 public records belonging to 41 BINs or 13 species from Gryllacrididae, 1493 public records belonging to 150 BINs or 656 species from Rhaphidophoridae (accessed on 1 Dec., 2021).

Much research has been done on Zhejiang katydid and related ensiferan groups (Wang and Tong 2014; Wu et al. 2014; Liu et al. 2018). Currently, 115 species of Tettigoniidae, 12 species of Gryllacrididae, and 18 species of Rhaphidophoridae have been recorded from Zhejiang Province, China (see Suppl. material 1). Here, we present the next step in building-up a DNA barcode reference library for the katydids, cave crickets, and leaf-rolling crickets from Zhejiang Province, China. These DNA barcodes can help greatly in flagging unusual specimens that merit more careful revision using morphological characters.

## ﻿Materials and methods

### ﻿Sampling of specimens

**Figure 1.**
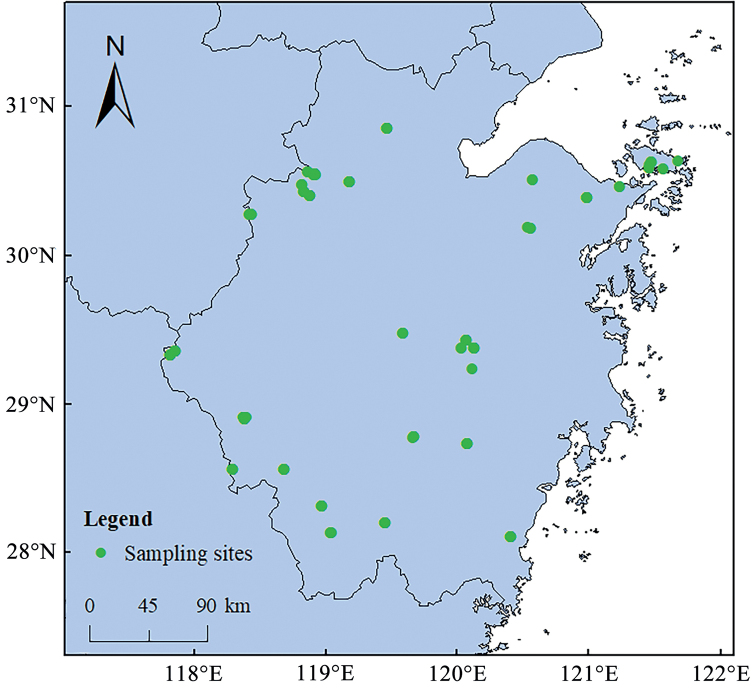
Sampling coverage of katydids, cave crickets, and leaf-rolling crickets in Zhejiang Province, China.

Collections were performed throughout Zhejiang Province, China in the period of 2011–2019. Collection information (Fig. 1) can be found in the BOLD system under the public dataset DS-ZJCK. All specimens were preserved in absolute ethanol and identified by Yizheng Zhao using morphological traits, i.e., body shape, pronotum, and genitalia (Gorochov and Le 2002; Liu and Kang 2007; Guo and Shi 2012; Di et al. 2014; Jiao et al. 2014; Shi and Wang 2015; Bian and Shi 2016; Feng et al. 2016; Qin et al. 2016; Shi et al. 2016; Qin et al. 2017; Zhu and Shi 2018; Liu et al. 2019, 2021).

### ﻿DNA extraction and COI barcode region sequencing

Total genomic DNA was extracted from hind legs of adults (*N* = 676) and nymphs (*N* = 5) using the Dneasy Blood and Tissue Kit (Tiangen Biotech, Beijing, China) according to the manufacturer’s specifications. The remainder of the specimen was retained as a voucher stored at the Katydids Lab of Hebei University, China. The COI barcode region was amplified with primers COBU (5´-TYT CAA CAA AYC AYA ARG ATA TTG G-3´) and COBL (5´-TAA ACT TCW GGR TGW CCA AAR AAT CA-3´) (Pan et al. 2006). PCR amplification reactions were performed as follows. The 50 µL of PCR mix contained 25 µL of Premix Taq (TaKaRa), 5 µL of each primer, 3 µL of templated DNA and 12 µL of ddH_2_O. The PCR cycling protocol included an initial denaturation at 94 °C for 3 min, followed by 35 cycles of denaturation at 94 °C for 30 s, annealing at 49 °C for 30 s, extension at 72 °C for 1 min, with a final extension at 72 °C for 8 min. All amplicons were sent to GENEWIZ (Tianjin, China) for bidirectional sequencing using ABI 3730XL DNA sequencers.

### ﻿Data analyses

Forward and reverse sequences were trimmed, edited, and assembled to produce a consensus barcode sequence using SeqMan Pro (DNA star, Inc., Madison, Wisconsin, USA) for each specimen. All COI-5P barcode sequences were examined for potential stop codons using Editseq (DNA star, Inc., Madison, Wisconsin, USA). All sequences were aligned by employing MUSCLE (codons) algorithm (Edgar, 2004) with default parameters in MEGA ver. 7.0 (Kumar et al. 2016). The resulting alignments were cropped to a length of 658 bp. The COI-5P barcode sequences, trace files, and voucher information (i.e., collection data, photograph, taxonomic assignment) for each specimen are available in the BOLD dataset DS-ZJCK. All sequences meeting required quality criteria (> 500 bp, < 1% Ns, no stop codon or contamination flag) were assigned to a BIN by the BOLD system (Ratnasingham and Hebert 2013). Taxon ID Tree, BIN discordance, genetic distance analysis, and Barcode Gap Analysis were performed using analytical tools in BOLD ver.4 on 1 Dec., 2021. The NJ tree was generated on BOLD with the Taxon ID tree tool using a Kimura-2-Parameter model, which is the mostly applied model in DNA barcoding studies (Hebert et al. 2003). The NJ tree was visualized using FigTree ver.1.4.4 (Rambaut 2018). BIN Discordance analysis on BOLD employs the Refined Single Linkage (RESL) algorithm for assigning barcode sequences to MOTUs independent of the BIN registry (Ratnasingham and Hebert 2013). There were four possible patterns of association between Linnaean species and Barcode Index Numbers (BINs), e.g., MATCH, SPLIT, MERGE, and MIXTURE. It should be noted that the BIN system is dynamic and dependent on the underlying data. Intraspecific distances and Barcode Gap Analysis could only be calculated for the 55 non-singleton species. The Barcode Gap Analysis provides mean and maximum intraspecific variations and a minimum genetic distance to the nearest-neighbour species (i.e., minimum interspecific distance).

In addition to BIN Discordance analysis, we also used other molecular delineation methods to delineate MOTUs. To minimize the risk of oversplitting (Talavera et al. 2013), the dataset was collapsed to retain only unique haplotypes. Four species delimitation approaches were employed: Assemble Species by Automatic Partitioning (ASAP) (Puillandre et al. 2021), jMOTU (Jones et al. 2011), General Mixed Yule Coalescent (GMYC) (Fujisawa and Barraclough 2013), and bPTP (Zhang et al. 2013). ASAP analysis was performed on the Web interface (https://bioinfo.mnhn.fr/abi/public/asap/asapweb.html) applying the K2P model, using default parameters (Puillandre et al. 2021). The jMOTU analysis was performed at cutoffs from 1 to 40 bp, covering a range between 0.15% and 6.08% divergence across the 658 bp COI-5P barcode. The General Mixed Yule Coalescent (GMYC) is a likelihood method for delimiting species, which tries to find the threshold between divergence events at the species level (modelled by a Yule process) and coalescent events between lineages within species (modelled by the coalescent). Both single-threshold GMYC (sGMYC) (Pons et al. 2006) and multi-threshold GMYC (mGMYC) (Monaghan et al. 2009) were computed. The best-fit nucleotide evolution model GTR+F+G4 was chosen by ModelFinder (Kalyaanamoorthy et al. 2017) under the Bayesian Information Criterion (BIC). The Bayesian Inference (BI) tree used for GMYC analysis was constructed using BEAST (Drummond and Rambaut 2007) using the Yule model and a constant clock. We checked runs for convergence and proper sampling of parameters [effective sample size (ESS) >200] using Tracer ver.1.7.1 (Rambaut et al. 2018). The BI tree was converted to the Newick format using FigTree ver.1.4.4 (Andrew 2016). The R package SPLITS (Ezard et al. 2009) was used for sGMYC and mGMYC analyses. The bPTP analysis models species formation events based on the number of substitutions in a given branch (Zhang et al. 2013). We used the BEAST tree created above to compare the generated outputs. The bPTP analysis was run using an online web server (https://species.h-its.org/ptp/) with default parameters except setting root tree, removing outgroup and MCMC generation = 500,000.

The results of different species delimitation methods were pairwise compared. Firstly, match ratio [2×N_match_/(N_A_+N_B_)] (Ahrens et al. 2016), where N_match_ is the number of molecularly delimited species using two different methods exactly matching, N_A_ and N_B_ is the number of delimited species by methods A and B, respectively. Secondly, taxonomic index of congruence [*C*_tax_(AB)= n(A∩B)/n(A⋃B)] (Miralles and Vences 2013), where A∩B represents the number of speciation events shared by methods A and B, and A⋃B represents the total number of speciation events inferred by method A and/or B. Thirdly, relative taxonomic resolving power index [*R*_tax_A = nA/n(A⋃B⋃C⋃D⋃E)] (Miralles and Vences 2013), where A, B, C, D, E represent the five species delimitation methods tested, nA represents the number of speciation events inferred by method A, and the denominator represents the cumulative number of speciation events inferred by all methods. Although large *R*_tax_ implies small type II error, it does not necessarily imply correct delimitations (i.e., can lead to over splitting) (Blair and Bryson 2017).

## ﻿Results

The COI-5P of 681 specimens of katydids, cave crickets, and leaf-rolling crickets were sequenced. Among these specimens, 601 (88.25%) specimens were identified to 69 morphospecies (formally described species that are typically defined by distinct morphological characters) and the remaining 80 specimens were only identified at genus level (Tables 1, 2 and 3). The number of specimens per species ranged from one (14 singletons) to 58 in *Gampsocleissinensis* Walker, 1869. Approximately half of these 69 morphospecies have five or more DNA barcodes. All sequences met the quality criteria (< 1% N and length > 500 bp) for BIN assignment. No insertions or deletions were observed.

### ﻿Removal of problematic specimens

**Figure 2.**
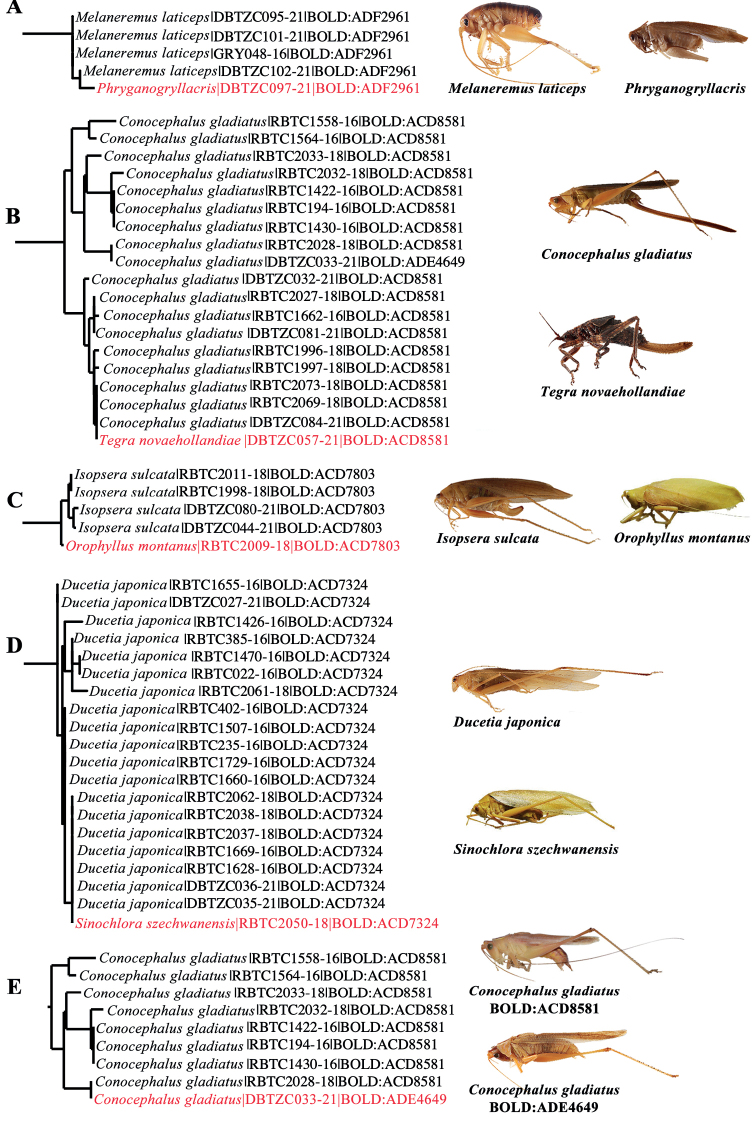
BIN discordance and the problems of interpreting potential NUMTs **A–E** represent the five cases of BIN discordance and individuals in red font represent individuals with potential NUMTs.

The preliminary “BIN Discordance” analysis (using BOLD ver.4 on 28 Dec., 2021) revealed five cases of merging, where each of the five BINs included two species from different genera or higher taxonomic taxa (Table 1). Species pairs in these five cases were distinctly morphologically different (Fig. 2). Five sequences located in apparently wrong positions on the NJ tree. To exclude contamination, DNA extraction from different leg and sequencing of these samples were repeated. Repeated experiments revealed that *Conocephalusgladiatus* Redtenbacher, 1891 DBTZC033-21 appeared to have resulted from experimental operation errors, and the remaining four cases could not be explained by contamination or lab errors. We had updated the sequences of *C.gladiatus*DBTZC033-21 prior to our analysis, and it clustered with other *C.gladiatus* specimens on the NJ tree. It sharing ADE4649 with *Diestramimaaustrosinensis* Gorochov, 1998 was the result of initial BIN assignment based on the previous incorrect sequence. Four records (*Orophyllusmontanus* Beier, 1954 RBTC2009-18, *Phryganogryllacris*DBTZC097-21, *Sinochloraszechwanensis* Tinkham, 1945 RBTC2050-18, and *Tegranovaehollandiae* Haan, 1843 DBTZC057-21) were highly likely COI-5P nuclear mitochondrial pseudogene (NUMT) intrusions and were excluded from our final dataset, since they each grouped separately from other individuals of the same species in the NJ tree (Fig. 5). Subsequent analyses focused on 677 barcode sequences, which were collapsed into 360 unique haplotypes. These records belong to three families, including Gryllacrididae (*N* = 35), Rhaphidophoridae (*N* = 23), and Tettigoniidae (*N* = 619).

**Table 1.** Results of the internal BIN discordance report for the five BINs of 83 specimens. # sequences have been resubmitted, * possibly NUMT coamplification.

BIN	Conflicting species	Taxonomic rank
ADE4649	*Diestramimaaustrosinensis* (6) *Conocephalusgladiatus*DBTZC033-21#	family
ACD8581	*Conocephalusgladiatus* (17) *Tegranovaehollandiae*DBTZC057-21*	subfamily
ACD7803	*Isopserasulcate* (4) *Orophyllusmontanus*RBTC2009-18*	subfamily
ACD7324	*Ducetiajaponica* (47) *Sinochloraszechwanensis*RBTC2050-18*	genus
ADF2961	*Melaneremuslaticeps* (4)*Phryganogryllacris*DBTZC097-21*	genus

### ﻿Genetic divergence

**Figure 3.**
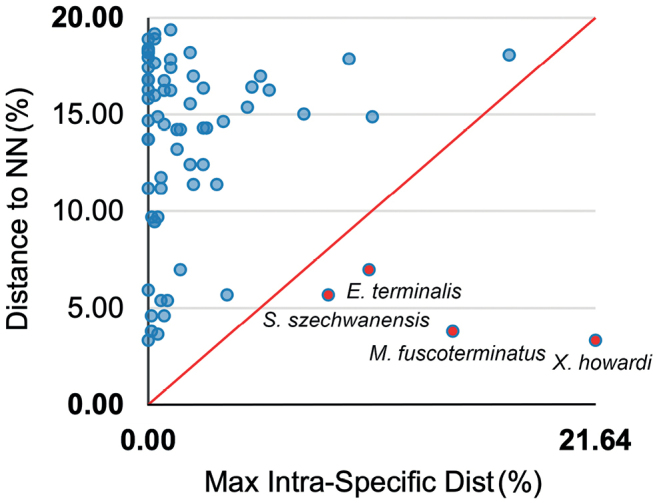
Scatter plot of maximum intraspecific distance and distance to nearest neighbour (NN). The four species to the right of the line represent a large intraspecific genetic distance.

Genetic distances for the resulting sequences were calculated in the BOLD System Distance Summary and Barcode Gap Analysis tools based on the K2P model. Table 4 provides sequence divergences (K2P) for differing levels of taxonomic affinity. The maximum intraspecific genetic distances (*I*_max_) of the 55 non-singleton species averaged 3.17% (range 0–21.64%), in which 24 species were above 2% (Table 2). Fourteen species are represented by only a single record, not allowing us to estimate intraspecific divergence. The genetic distance to the nearest neighbour (DNN) averaged 13.14% (ranging 3.31–19.38%), with the minimum nearest-neighbour distance occurring between *Xizicuslaminatus* Shi, 2013 and *Xizicushowardi* Tinkham, 1956 (Table 2). Not a single haplotype was shared between species within our DNA barcode library. A barcode gap was present in 51 of 55 (92.73%) non-singleton species. Intraspecific distances were inflated by the presence of very high variation within some taxa, resulting in no significant barcode gaps (Fig. 3). The maximum intraspecific distance was higher than its nearest-neighbour distance in four species, including *Elimaeaterminalis* Liu, 1993, *Melaneremusfuscoterminatus* Brunner von Wattenwyl, 1888, *Sinochloraszechwanensis*, and *Xizicushowardi* (Table 1, Fig. 3). Eighteen of 24 species with deep intraspecific divergence (K2P model, *I*_max_ > 2%) were split into two or more BINs (Table 2). Interestingly, *Ruspoliadubia* Redtenbacher, 1891 were also split into two BINs, although the intraspecific divergence was relatively low (*I*_max_ = 1.55%).

**Table 2.** BIN assignments and genetic divergence of 68 morphospecies. BIN, Barcode Index Number; *N*, number of barcodes per BIN; *I*_mean_, mean intraspecific distance; *I*_max_, maximum intraspecific distance; DNN, distance to nearest neighbour; species in bold and labelled* *I*_max_> DNN. Singletons are labeled as N/A and could not be evaluated.

Species	BIN (*N*)	*I*_mean_	*I*_max_	Nearest Neighbour	DNN
Gryllacrididae					
*Apotrechusbilobus*	ADF4059 (3)	0.31	0.46	*Eugryllacriselongata* DBTZC100-21	14.88
*Capnogryllacrismelanocrania*	ADF2751 (1) AEJ4972 (1) ADF2750 (2) AEJ9445 (2)	3.21	5.01	*Eugryllacriselongata* GRY018-16	16.4
*Eugryllacriselongata*	AEK0366 (1) ADF4811 (5)	3.63	10.84	*Apotrechusbilobus* DBTZC103-21	14.88
*Homogryllacrisanelytra*	ADF3866 (3)	0.83	1.09	*Phryganogryllacrisxiai* GRY040-16	17.42
Melaneremusfuscoterminatus*	ADF2959 (1) ADF2960 (1)	14.73	14.73	*Melaneremuslaticeps* DBTZC101-21	3.78
*Melaneremuslaticeps*	ADF2961 (4)	0.08	0.15	*Melaneremusfuscoterminatus* GRY049-16	3.78
*Metriogryllacrispermodesta*	ADF4959 (1)	N/A	0	*Phryganogryllacrisxiai* GRY040-16	18.4
*Phryganogryllacrissuperangulata*	ADF3568 (5)	0.15	0.31	*Capnogryllacrismelanocrania* DBTZC078-21	18.92
*Phryganogryllacrisxiai*	ADF3457 (1)	N/A	0	*Homogryllacrisanelytra* DBTZC096-21	17.42
Rhaphidophoridae					
*Diestramimaaustrosinensis*	ADE4649 (6)	0.3	0.61	*Diestramimabrevis* DBTZC116-21	5.39
*Diestramimabrevis*	AEJ2460 (5)	0.37	0.93	*Diestramimaaustrosinensis* DBTZC054-21	5.39
*Gymnaetoidestestaceus*	AEJ5191 (3)	1.45	2.18	*Tachycinesmeditationis* DBTZC126-21	11.39
*Microtachycineselongatus*	AEJ2738 (2)	0.62	0.62	*Tachycinesmeditationis* DBTZC130-21	11.75
*Tachycinesmeditationis*	AEJ6894 (1) AEK0279 (2) AEJ9615 (4)	1.77	3.31	*Gymnaetoidestestaceus* DBTZC123-21	11.39
Tettigoniidae					
*Atlanticusinterval*	ADE2184 (3)	0.72	1.08	*Holochloravenusta* RBTC2022-18	19.38
*Conocephalusbidentatus*	ADB6577 (1)	N/A	0	*Conocephalusmaculatus* RBTC1645-16	18.31
*Conocephalusgladiatus*	ADE4649 (1) ACD8581 (17)	1.06	2.18	*Conocephalusmaculatus* RBTC1645-16	16.97
*Conocephalusmaculatus*	ACD2116 (1) ADB5579 (2)	3.62	5.43	*Conocephalusgladiatus* DBTZC032-21	16.97
*Conocephalusmelaenus*	ACD4634 (20)	0.1	0.31	*Conocephalusgladiatus* DBTZC032-21	17.65
*Defloritadeflorita*	ADB3725 (14)	0.79	2.67	*Hemielimaeachinensis* RBTC2067-18	16.35
*Ducetiajaponica*	ACD7324 (47)	1.23	2.67	*Kuwayamaeabrachyptera* DBTZC001-21	14.3
*Elimaeaannamensis*	ADE1944 (9)	0.46	1.55	*Elimaeaterminalis* RBTC2046-18	6.98
*Elimaeacheni*	ADB3480 (13)	0.09	0.46	*Elimaeananpingensis* DBTZC006-21	9.69
*Elimaeananpingensis*	ADB3475 (12)	0.06	0.17	*Elimaeacheni* DBTZC026-21	9.69
Elimaeaterminalis*	ADB3392 (3) ADB3394 (3)	6.35	10.68	*Elimaeaannamensis* RBTC1668-16	6.98
*Euconocephalusnasutus*	ACD6726 (2)	1.39	1.39	*Ruspoliadubia* RBTC1561-16	14.22
*Euxiphidiopsiscapricercus*	ADE2467 (1)	N/A	0	*Gampsocleissinensis* RBTC1223-16	18.21
*Gampsocleissinensis*	AAY1322 (58)	0.87	2.03	*Euxiphidiopsiscapricercus* HLXX121-16	18.21
*Grigorioracheni*	ADE0541 (7)	0.79	1.39	*Sinocyrtaspisbrachycerca* PSM013-19	13.2
*Hemielimaeachinensis*	ADB3478 (16) AEJ5565 (2) ADE2233 (4)	1.51	3.63	*Elimaeananpingensis* DBTZC006-21	14.64
*Hexacentrusjaponicus*	ACD8277 (4) ADM2486 (4)	1.53	2.66	*Hexacentrusunicolor* BHC097-18	12.42
*Hexacentrusunicolor*	ACD7247 (36)	0.65	2.03	*Hexacentrusjaponicus* BHC079-15	12.42
*Holochlorajaponica*	ADE1373 (6)	0.16	0.31	*Holochloravenusta* RBTC2063-18	9.45
*Holochloravenusta*	ADB6143 (12)	0.05	0.31	*Holochlorajaponica* RBTC1717-16	9.45
*Isopseradenticulata*	AEJ6400 (1) ADE1596 (5) ADB3788 (7) ACD5193 (9)	5.96	9.72	*Defloritadeflorita* RBTC216-16	17.88
*Isopserafurcocerca*	ADB4481 (5)	0	0	*Paraxantiahuangshanensis* RBTC1295-16	17.96
*Isopserasulcate*	ACD7803 (4)	0.18	0.31	*Isopserafurcocerca* RBTC196-16	19.17
*Kuwayamaeabrachyptera*	AEJ7401 (1) AEK2062 (1) AEK1896 (3)	1.81	2.82	*Ducetiajaponica* DBTZC015-21	14.3
*Mecopodaniponensis*	AAF0977 (1) ACD8152 (18)	1.09	7.53	*Diestramimaaustrosinensis* DBTZC054-21	15.02
*Mirolliabispina*	ADB4146 (3)	0.61	0.77	*Mirolliabispinosa* RBTC406-16	4.61
*Mirolliabispinosa*	ADB4148 (3)	0.1	0.15	*Mirolliabispina* RBTC237-16	4.61
*Nigrimaculaparaquadrinotata*	ACD6675 (1)	N/A	0	*Grigorioracheni* HLXX071-16	15.82
*Palaeoagraeciaascenda*	ACD8365 (4)	0	0	*Mecopodaniponensis* RBTC2086-18	16.8
*Paraxantiahuangshanensis*	ADB6578 (1)	N/A	0	*Nigrimaculaparaquadrinotata* HLXX059-16	16.76
*Phaneropterafalcata*	AAL2811 (2)	0.31	0.31	*Kuwayamaeabrachyptera* DBTZC012-21	15.98
*Phaneropteranigroantennata*	ACD4406 (2)	0.77	0.77	*Ducetiajaponica* RBTC397-16	14.49
*Phyllomimusklapperichi*	ADM7559 (1) ADB9999 (4) ADB4775 (6)	10.3	17.47	*Ducetiajaponica* RBTC397-16	18.08
*Pseudocosmeturafengyangshanensis*	ADW0286 (1)	N/A	0	*Sinocyrtaspisbrachycerca* PSM014-19	11.19
*Pseudokuzicuspieli*	ACD4648 (1)	N/A	0	*Teraturamegafurcula* HLXX099-16	13.71
*Pseudorhynchusconcisus*	ADB6233 (7)	0.37	1.08	*Pyrgocoryphaparva* BOCON142-16	16.24
*Pyrgocoryphaparva*	ADC0410 (3)	0.51	0.77	*Pseudorhynchusconcisus* DBTZC059-21	16.24
*Qinlingeabrachystylata*	ADB4056 (1)	N/A	0	*Ruidocollaristruncatolobata* RBTC1677-16	18.9
*Ruidocollaristruncatolobata*	ACD6433 (15) ADB6075 (5)	2.2	5.85	*Ducetiajaponica* DBTZC015-21	16.25
*Ruspoliadubia*	ACD5503 (1) ADE5391 (3)	1.09	1.55	*Euconocephalusnasutus* RBTC1705-16	14.22
*Ruspolialineosa*	ACD5257 (26)	0.79	2.03	*Ruspoliadubia* RBTC1649-16	15.55
*Sinochloralongifissa*	AEJ1447 (1) ADB3789 (34)	1.1	3.81	*Sinochloraszechwanensis* DBTZC067-21	5.68
*Sinochlorasinensis*	ACD4415 (1)	N/A	0	*Sinochloraszechwanensis* DBTZC038-21	5.93
Sinochloraszechwanensis*	ACI0121 (2) ADB3463 (4)	4.61	8.71	*Sinochloralongifissa* DBTZC039-21	5.68
*Sinocyrtaspisbrachycerca*	ADX3437 (4)	0.41	0.61	*Pseudocosmeturafengyangshanensis* PSM017-19	11.19
*Tegranovaehollandiae*	ADB5353 (10)	0.39	1.08	*Ducetiajaponica* RBTC249-16	17.85
*Teraturamegafurcula*	ACD5306 (1)	N/A	0	*Pseudokuzicuspieli* RBTC411-16	13.71
*Tettigoniachinensis*	ACD6622 (8)	0.32	0.77	*Hemielimaeachinensis* DBTZC092-21	16.74
*Xiphidiopsisgurneyi*	ADE1670 (2)	0	0	*Grigorioracheni* HLXX074-16	16.27
*Xizicusbiprocerus*	ADE1374 (1)	N/A	0	*Pseudokuzicuspieli* RBTC411-16	14.68
*Xizicusconcavilaminus*	ADB3332 (3)	0.31	0.46	*Xizicuslaminatus* HLXX037-16	3.63
Xizicushowardi*	AEJ3139 (1) ADB5688 (10) ACD5539 (3) ADE3141 (4)	6.13	21.64	*Xizicuslaminatus* HLXX037-16	3.31
*Xizicuslaminatus*	ADB5868 (1)	N/A	0	*Xizicushowardi* RBTC1648-16	3.31
*Xizicusszechwanensis*	ADE0823 (2) ADB3348 (9)	1.55	4.8	*Xizicushowardi* DBTZC013-21	15.37

### ﻿Barcode index numbers (BINs) assignment and species delimitation

For the final dataset, 677 COI-5P records were assigned to 118 BINs that belong to 90 putative taxa. Among these, 68 corresponded to morphospecies, while another 22 belonged to a unique BIN that was currently only identified at genus level and highly likely to represent an unrecognized species. Of 68 morphospecies defined by morphology, a total of 49 contained only a single BIN, while 19 were represented by multiple BINs (Table 2). The average number of BINs per species in “split” cases was 2.53, ranging from 2 to 4. Two BINs were found in each of 12 species: *Eugryllacriselongata* Bian & Shi, 2016 (AEK0366, ADF4811), *Melaneremusfuscoterminatus* (ADF2959, ADF2960), *Conocephalusgladiatus* (ADE4649, ACD8581), *Conocephalusmaculatus* Le Guillou, 1841 (ACD2116, ADB5579), *Elimaeaterminalis* (ADB3392, ADB3394), *Hexacentrusjaponicus* Karny, 1907 (ACD8277, ADM2486), *Mecopodaniponensis* Haan, 1843 (AAF0977, ACD8152), *Ruidocollaristruncatolobata* Brunner von Wattenwyl, 1878 (ACD6433, ADB6075), *Ruspoliadubia* (ACD5503, ADE5391), *Sinochloralongifissa* Matsumura & Shiraki, 1908 (AEJ1447, ADB3789), *Sinochloraszechwanensis* (ACI0121, ADB3463), and *Xizicusszechwanensis* Tinkham, 1944 (ADE0823, ADB3348). Three BINs were found in each of four species: *Tachycinesmeditationis* Würmli, 1973 (AEJ6894, AEK0279, AEJ9615), *Hemielimaeachinensis* Brunner von Wattenwyl, 1878 (ADB3478, AEJ5565, ADE2233), *Kuwayamaeabrachyptera* Gorochov & Kang, 2002 (AEJ7401, AEK2062, AEK1896), and *Phyllomimusklapperichi* Beier, 1954 (ADM7559, ADB9999, ADB4775). Four BINs were found in each of three species: *Capnogryllacrismelanocrania* Karny, 1929 (ADF2751, AEJ4972, ADF2750, AEJ9445), *Isopseradenticulata* Ebner, 1939 (AEJ6400, ADE1596, ADB3788, ACD5193), and *Xizicushowardi* (AEJ3139, ADB5688, ACD5539, ADE3141) (Table 1). Furthermore, 79 sequenced specimens that only identified at genus level were allocated to 22 BINs. The interim species names of these unidentified specimens consisted of the genus name plus a corresponding BIN ID, such as *Bulbistridulous*BOLD:ADB3431. Specimens of five genera were each assigned to a unique BIN: *Bulbistridulous*BOLD:ADB3431, *Conanalus*BOLD:ADB5687, *Hexacentrus*BOLD:ADB5446, *Phryganogryllacris*BOLD:ADF3837, and *Prohimerta*BOLD:ADB4147, suggesting that each belongs to a single species. In contrast, specimens of the remaining three genera were heterogeneous and split into two or more BINs: *Elimaea*BOLD:ADE1399, ADM8940, and ADB3477; *Atlanticus*BOLD:ADB5602, ADB6974, ADR7192, ADE2402, ADB3445, ADE1821, and ADB3462; *Kuwayamaea*BOLD:ADB4962, ADE2183, ADE1620, ADB6899, ADB4961, ADB5240, and ADB4960.

**Table 3.** BIN assignments of 79 specimens identified only to genus level. BIN, Barcode Index Number; ***N***, number of barcodes per BIN.

Taxon	BIN (*N*)
*Atlanticus*	ADB5602 (1), ADB6974 (1), ADR7192 (1), ADE2402 (1), ADB3445 (2), ADE1821 (2), ADB3462 (3)
*Bulbistridulous*	ADB3431 (1)
*Conanalus*	ADB5687 (1)
*Elimaea*	ADE1399 (1), ADM8940 (1), ADB3477 (1)
*Hexacentrus*	ADB5446 (2)
*Kuwayamaea*	ADB4962 (1), ADE2183 (1), ADE1620 (13), ADB6899 (27), ADB4961 (4), ADB5240 (4), ADB4960 (6)
*Phryganogryllacris*	ADF3837 (4)
*Prohimerta*	ADB4147 (1)

The NJ tree was employed to assess support for detected BINs, not to reconstruct the phylogenic relationships. The NJ tree showed the majority of non-singleton species and BINs were recovered as monophyletic (Fig. 5). All BIN species represented by two or more specimens, except ADE4649, formed a monophyletic lineage. High intraspecific divergence values also reflected deep splits in the NJ tree. All non-singleton morphospecies are clearly distinguishable through COI-5P, forming non-overlapping clades except for several species with deep intraspecific divergence exceeding DNN, namely *Elimaeaterminalis* (*I*_max_ = 10.68%, DNN = 6.98), *Melaneremusfuscoterminatus* (*I*_max_ = 14.73%, DNN = 3.78%), *Sinochloraszechwanensis* (*I*_max_ = 8.71%, DNN = 5.68%) and *Xizicushowardi* (*I*_max_ = 21.64%, DNN = 3.31%) (Fig. 5, Table 2).

**Tables 4.** Kimura 2 Parameter sequence divergence at each taxonomical level.

Distance class	n	Taxa	Comparisons	Min Dist (%)	Mean Dist (%)	Max Dist (%)
Intraspecific	585	55	6407	0.00	1.44	21.49
Congeners	314	13	2439	3.29	15.19	24.30
Confamilial	598	3	139568	11.12	22.66	34.59

ASAP analysis identified 99 MOTUs with an asapscore of 9.00 (Fig. 5). jMOTU analysis delimited 101 at a 20 bp (3%) cut-off divergence (Fig. 5). The GMYC single-threshold method estimated 105 MOTUs, while the GMYC method with multiple thresholds delimited 132 MOTUs (Fig. 5). The bPTP analysis delimited 119 and 120 MOTUs based on the maximum likelihood and highest Bayesian supported analyses, respectively (Fig. 5).

**Figure 4.**
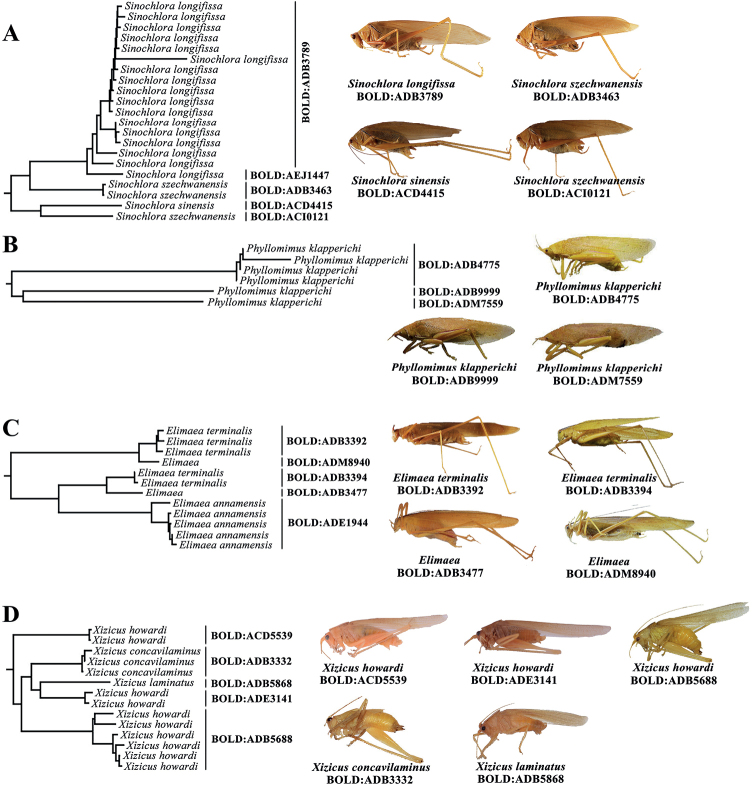
Examples of species split into more than one BIN **A–D** represent *Sinochloraszechwanensis* split into 2 BINs, *Phyllomimusklapperichi* split into 3 BINs, *Elimaeaterminalis* split into 2 BINs, and *Xizicushowardi* split into 3 BINs.

**Figure 5.**
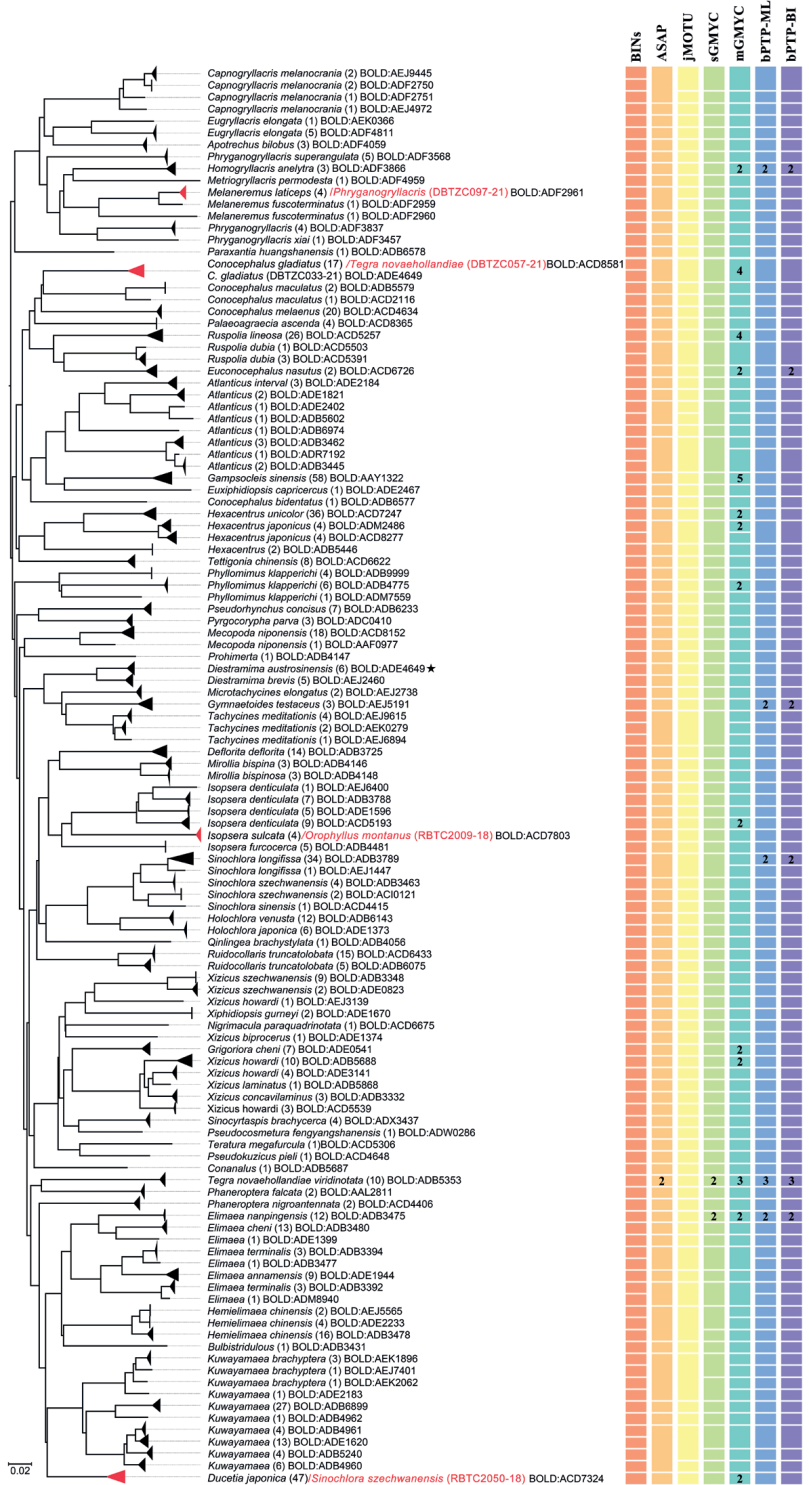
BOLD TaxonID Tree based on K2P distances and species delimitation results based on COI-5P sequences. The four barcode sequences marked by red are highly likely NUMTs and excluded from the species delimitation analyses. The MOTUs created by each delimitation algorithm are represented as squares on the right. The number within the rectangles indicates the number of MOTUs; no number indicates a single MOTU.

*Capnogryllacrismelanocrania* showed deep intraspecific divergence (*I*_max_ = 5.01%), and was split into four BINs (ADF2751, AEJ4972, ADF2750, AEJ9445), and these four BINs formed nearest-neighbour clusters. All species delimitation methods treated *C.melanocrania* as three MOTUs (ADF2750 and AEJ9445 were placed in a single MOTU, while ADF2751 and AEJ4972 were each placed in their own MOTU), except for ASAP which placed all specimens of *C.melanocrania* in two MOTUs. *Eugryllacriselongata* showed deep intraspecific divergence (Max Intra-Sp = 10.68%), and was split into two BINs (AEK0366, ADF4811). All species delimitation methods treated *E.elongata*AEK0366 and ADF4811 as two separate MOTUs. *Melaneremusfuscoterminatus* showed deep intraspecific divergence (*I*_max_ = 14.73%) and was split into two BINs (ADF2959 and ADF2960). The nearest neighbour of the *M.laticeps*ADF4959 clade was *M.fuscoterminatus*ADF2959, followed by *M.fuscoterminatus*ADF2960. All species delimitation methods suggested *M.fuscoterminatus*ADF2959 should be treated as a separate MOTUs. ASAP treated *M.fuscoterminatus*ADF2959 and *M.laticeps*ADF4959 as one MOTU. *Conocephalusmaculatus* showed deep intraspecific divergence (Max Intra-Sp = 5.43%), and was split into two BINs (ACD2116, ADB5579). All species delimitation methods treated *E.elongata*ACD2116 and ADB5579 as two separate MOTUs. *Sinochloraszechwanensis* showed deep intraspecific divergence (*I*_max_ = 8.71%), while no barcode gap was present in *S.szechwanensis* and *S.longifissa* (Table 2). Three *Sinochlora* species formed two clades: one was composed of specimens identified as *S.szechwanensis* (ADB3463) and *S.sinensis*, and the other was composed of *S.szechwanensis* (ACI0121) and *S.longifissa* (Fig. 4). *Phyllomimusklapperichi* showed deep intraspecific divergence (*I*_max_ = 17.47%), and was split into three BINs (ADM7559, ADB9999, and ADB4775) (Fig. 4), reflecting three distinctly different subclusters of the *P.klapperichi* cluster on the NJ tree. All species delimitation methods split *P.klapperichi* into three MOTUs, except for mGMYC that split *P.klapperichi* into four MOTUs. *Elimaeaterminalis* showed deep intraspecific divergence (*I*_max_ = 10.68%) and was split into two BINs (ADB3392 and ADB3394). Two *E.terminalis*BINs corresponded to two clades in the NJ tree: one contained three *E.terminalis*ADB3392 specimens and was sister to *Elimaea*ADM8940, whereas the other contained three *E.terminalis*ADB3394 specimens, which was sister to *Elimaea*ADB3477 and *Elimaeaannamensis* Hebard, 1922 (Fig. 4). All species delimitation methods treated two *E.terminalis*BINs as separate MOTUs. Both *Xizicushowardi* (*I*_max_ = 21.64%) and *X.szechwanensis* (Max Intra-Sp = 4.8%) showed deep intraspecific divergence, and were split into four BINs (ACD5539, ADE3141, ADB5688, and AEJ3139) and two BINs (ADB3348 and ADE0823), respectively. Five *Xizicus* species formed three clades on the NJ tree: the first composed by specimens identified as *X.concavilaminus*, *X.laminatus*, and three *X.howardi*BINs (ACD5539, ADE3141, ADB5688); the second composed of all *X.szechwanensis* specimens and *X.howardi*AEJ3139, and the third composed of only a single *X.biprocerus*. All species delimitation methods except mGMYC revealed consistent results with BIN assignments. mGMYC split *X.howardi*ADB5688 as two MOTUs (Figs 4, 5). Therefore, *X.howardi* in Zhejiang might be a species complex of at least four species. Detailed comparative analyses of additional specimens was needed to evaluate the taxonomic status of *X.howardi*. Although *Ruspoliadubia* (*I*_max_ = 1.55%) was split into two BINs (ACD5503, ADE5391), all species delimitation methods treated *R.dubia* as a single MOTU. *Tachycinesmeditationis* (*I*_max_ = 3.31%) was split into three BINs (AEJ6894, AEK0279, AEJ9615). mGMYC, bPTP-ML, bPTP-BI approaches agreed on the subdivision of *T.meditationis*BINs while ASAP, jMOTU, and sGMYC analyses treated *T.meditationis* as a single MOTU. A BIN was assigned to the Match category when all of its specimens were assigned to single MOTU. Fifty-five out of 90 species-level taxa were recovered by all species delineation methods, suggesting that they may be a single species. *R*_tax_ values ranged from 0.71 for ASAP to 0.94 for mGMYC (Table 5), suggesting that mGMYC may overestimate the number of species. *C*_tax_ values between different species delimitation methods ranged from 0.75 (ASAP vs. mGMYC) to 0.99 (bPTP-ML vs. bPTP-BI), whereas, match ratios ranged from 0.65 (ASAP vs. mGMYC) to 0.99 (bPTP-ML vs. bPTP-BI) (Table 5).

**Tables 5.** Calculation of match ratio, taxonomic index of congruence (*C*_tax_), and relative taxonomic resolving power index (*R*_tax_) for different species delimitation methods. The lower triangle shows *C*_tax_, and the upper triangle shows Match ratio.

	BIN	ASAP	jMOTU	sGMYC	mGMYC	bPTP -ML	bPTP-BI
**BIN**		0.76	0.82	0.79	0.73	0.87	0.86
**ASAP**	0.82		0.93	0.91	0.65	0.77	0.76
**jMOTU**	0.85	0.96		0.91	0.70	0.81	0.80
**sGMYC**	0.89	0.94	0.94		0.71	0.82	0.81
**mGMYC**	0.81	0.75	0.76	0.79		0.80	0.81
**bPTP-ML**	0.91	0.83	0.85	0.88	0.84		0.99
**bPTP-BI**	0.90	0.82	0.84	0.87	0.85	0.99	
**Mean C_tax_**	0.85	0.85	0.85	0.86	0.78	0.84	0.86
**R_tax_**	0.84	0.71	0.72	0.75	0.94	0.85	0.86
**Species**	118	99	101	105	132	119	120

## ﻿Discussion

In the past several hundred years, species diagnostics have been traditionally based on morphological characterizations. Morphology-based specimen identification is time consuming and requires high levels of taxonomic expertise. Compared with traditional taxonomy, DNA barcoding is a fast and inexpensive method for species identification. Numerous studies have revealed cryptic species using DNA barcodes (Kondo et al. 2016; Lassance et al. 2019; Farkas et al. 2020). However, using only DNA barcodes may lead to classification errors, and it is important to combine morphology and barcodes.

The utility of DNA barcoding heavily depends on the taxonomic coverage of an associated DNA barcode reference library. Barcode libraries are generally assembled with two main objectives in mind: specimen identification and aiding species discovery/delimitation (Knebelsberger et al. 2014; Blagoev et al. 2016; Khamis et al. 2017; Ashfaq et al. 2019; Delrieu-Trottin et al. 2019; D’Ercole et al. 2021). The significant increase in studies of specific insect taxa using DNA barcodes in recent years, especially in some regions, has laid the foundations for building a comprehensive library of DNA barcodes at the continental-scale (D’Ercole et al. 2021; Dincă et al. 2021; Pesic et al. 2021). Only a few barcode studies of katydids and related ensiferan groups have been conducted in China, South Korea, Central Europe (Guo et al. 2016; Hawlitschek et al. 2017; Zhou et al. 2019; Kim et al. 2020). Our study provided 677 COI-5P barcode sequences, including 68 morphospecies and 80 specimens only identified to genus level.

BIN sharing between different species might be explained by mitochondrial introgression following hybridization, recent divergence with or without incomplete lineage sorting, inadequate taxonomy, misidentification (Geiger et al. 2021). One large-scale study for European Lepidoptera showed that more than half (58.6%) of the detected cases of non-monophyletic species are likely to be due to operational factors such as misidentification, oversplitting of species, overlooked synonymies or potential cryptic species (Mutanen et al. 2016). For the DNA barcode library of Central and Northern European Odonata, six of 31 BINs containing records of mixed taxonomic annotations conflict at generic levels, which is most likely due to misidentification, sample mix-up in the laboratory, sample number mix-up of specimens, or nomenclatural changes not applied to all affected datasets in BOLD (Geiger et al. 2021). Our previously mentioned example of BIN sharing between *Conocephalusgladiatus*DBTZC033-21 and *Diestramimaaustrosinensis* was caused by a sample confusion. The accuracy of DNA barcoding can be severely impacted when there are atypical NUMTs that lack the characteristic mutations (including in-frame stop codons and indels), which were difficult to identify and remove from the barcode dataset. NUMTs are rarely reported in DNA barcoding studies, despite a fairly frequent abundance across various insect groups (Hausberger et al. 2011; Jordal and Kambestad 2014; Hawlitschek et al. 2017). Our study also revealed four records shared with other species, which were highly likely the erroneous amplification of nonfunctional nuclear copies of COI-5P. The specimens of different species were admixed in a single cluster on the NJ tree, which often arises as the result of misidentification, contamination, or NUMTs (Mutanen et al. 2016). Previous studies found that NUMTs are coamplified using universal primers LCO1490/HCO2198, even across families: *Anabrussimplex* (Tettigoniidae) vs. *Schistocercaamericana* (Acrididae) (Moulton et al. 2010). Many NUMTs not having stop codons or indels may represent mitochondrial heteroplasmy, but this is a highly unusual phenomenon in insects (Jordal and Kambestad 2014).

Our analyses revealed 19 of 55 non-singleton morphospecies (34.55%) with multiple BINs. Most of these intraspecific BINs formed nearest-neighbour clusters to each other, reflecting the discrimination of geographical subclades within a currently recognized species. Previous studies have shown that BINs provide a very good reflection of classical taxonomy (Hausmann et al. 2013). For example, our prior study has shown a three-quarter species-BIN correspondence in katydids from China (Zhou et al. 2019). Species with BIN splits and high divergences are likely to represent a cryptic species complex (Ashfaq et al. 2019). Likewise, high levels of ‘intraspecific’ barcode variation also reflect overlooked species, but there is no fixed level of divergence that indicates species status (Huemer et al. 2020). Although the presence of a barcoding gap, intraspecific variation threshold, or monophyly of each putative species are sufficient conditions to ensure specimen correct identification, these are not essential criteria (Meyer and Paulay 2005; Yang and Rannala 2017).

Applying multiple species delimitation methods to the same dataset can provide a more reliable picture of species-level clustering. We obtained more MOTUs based on both the distance-based species-delimitation (ASAP, jMOTU) and the phylogeny-based methods (GMYC and bPTP) than the number of morphospecies. Several species with deep intraspecific divergence were split into more than one MOTU, and most of these additional BINs formed nearest-neighbour subclusters on the NJ tree. It was worth exploring the large intraspecific genetic distances for the same species although they were clustered together. Inconsistencies in delimitation results occur frequently as the result of different species delimitation methods. The mGMYC analysis produced a considerably higher number of MOTUs than other methods. *R*_tax_ values ranged from 0.71 for ASAP to 0.94 for mGMYC (Table 5), suggesting that mGMYC may overestimate the number of species. The performance of phylogeny-based methods is sensitive to multiple factors, such as general phylogenetic history, sampling intensity, DNA sequence length, speciation rate, and differences of effective population size among species (Esselstyn et al. 2012). The number of species can be underestimate or overestimate with ancestral polymorphism (Esselstyn et al. 2012), but previous studies showed that the sGMYC performs better than mGMYC (Talavera et al. 2013). Three indicators (Match ratio, *C*_tax_, and *R*_tax_) suggest that different species definition methods also diverge in terms of the location of species boundaries (Miralles and Vences 2013). Concordance among results of different species delimitation methods revealed that both *P.klapperichi* and *E.annamensis* may contain undocumented cryptic species. At present, we speculate that this is due to allopatric isolation due to the mountainous barriers between the samples. Despite the lack of clear morphological differentiation, the geographically and genetically distinct clusters suggest the existence of cryptic diversity. Therefore, our study indicates that the diversity of katydids, cave crickets, and leaf-rolling crickets in Zhejiang Province is slightly higher than the currently accepted taxonomy would suggest. The concordance among different species delimitation methods often implies higher reliability and should be used as primary taxonomic hypotheses that are subsequently tested with other types of data as part of an integrative taxonomic framework (Fujita et al. 2012; Blair and Bryson 2017).

## ﻿Conclusions

Our DNA barcode library represents an important step for the molecular characterization of katydids, cave crickets, and leaf-rolling crickets in Zhejiang, China. Although some specimens still lack a Linnean name, their BIN assignments are treated as putative species in ecology, conservation biology and other biodiversity research (Sharkey et al. 2021). The number of detected BINs higher than traditionally accepted species suggests that DNA barcoding will complement morphology-based taxonomic system by revealing overlooked species complexes (Schmidt et al. 2015). The consensus delimitation scheme yielded 55 MOTUs, each of which may be a single species. Only three species (*I*_max_ > DNN) failed to be identified as monophyletic (e.g., *Elimaeaterminalis*, *Sinochloraszechwanensis*, and *Xizicushowardi*), so we speculate that these may be species complexes. If a species is split into two or more MOTUs implying cryptic diversity, then the number of katydid species in Zhejiang may be more than what is currently identified. However, prior to formal taxonomic changes, results should be subsequently tested using an integrative approach. This Barcode library was effective in assigning newly encountered specimens to either one or a few closely allied species. We expect it to be useful for future katydid taxonomic and conservation work.

## Supplementary materials

Supplementary material 1 List of species of the family Tettigoniidae, Rhaphidophoridae, and Gryllacrididae in Zhejiang Province, China Data type species data https://binary.pensoft.net/file/753158This dataset is made available under the Open Database License (http://opendatacommons.org/licenses/odbl/1.0/). The Open Database License (ODbL) is a license agreement intended to allow users to freely share, modify, and use this Dataset while maintaining this same freedom for others, provided that the original source and author(s) are credited.Yizheng Zhao, Hui Wang, Huimin Huang, Zhijun Zhou
